# A comparison of one-year treatment utilization for shoulder osteoarthritis patients initiating care with non-orthopaedic physicians and orthopaedic specialists

**DOI:** 10.1186/s12891-018-2268-3

**Published:** 2018-09-27

**Authors:** Sarah B Floyd, Cole G Chapman, Ellen Shanley, Lauren Ruffrage, Eldon Matthia, Peter Cooper, John M Brooks

**Affiliations:** 1 Department of Health Services Policy and Management, University of South Carolina, Columbia, SC USA; 2 Center for Effectiveness Research in Orthopaedics, Greenville, SC USA; 3 ATI Physical Therapy, Greenville, SC USA; 4 University of South Carolina School of Medicine Greenville, Greenville, SC USA; 5 Arnold School of Public Health, University of South Carolina, 915 Greene St., Suite 303C, Columbia, SC 29208 USA

**Keywords:** Shoulder, Osteoarthritis, Treatment, Surgery, Injections, Physical therapy

## Abstract

**Background:**

In this paper we investigate patients seeking care for a new diagnosis of shoulder osteoarthritis (OA) and the association between a patient’s initial physician specialty choice and one-year surgical and conservative treatment utilization.

**Methods:**

Using retrospective data from a single large regional healthcare system, we identified 572 individuals with a new diagnosis of shoulder OA and identified the specialty of the physician which was listed as the performing physician on the index shoulder visit. We assessed treatment utilization in the year following the index shoulder visit for patients initiating care with a non-orthopaedic physician (NOP) or an orthopaedic specialist (OS). Descriptive statistics were calculated for each group and subsequent one-year surgical and conservative treatment utilization was compared between groups.

**Results:**

Of the 572 patients included in the study, 474 (83%) received care from an OS on the date of their index shoulder visit, while 98 (17%) received care from a NOP. There were no differences in baseline patient age, gender, BMI or pain scores between groups. OS patients reported longer symptom duration and a higher rate of comorbid shoulder diagnoses. Patients initiating care with an OS on average received their first treatment much faster than patients initiating care with NOP (16.3 days [95% CI, 12.8, 19.7] vs. 32.3 days [95% CI, 21.0, 43.6], Z = 4.9, *p* < 0.01). Additionally, patients initiating care with an OS had higher odds of receiving surgery (OR = 2.65, 95% CI: 1.42, 4.95) in the year following their index shoulder visit.

**Conclusions:**

Patients initiating care with an OS received treatment much faster and were treated with more invasive services over the year following their index shoulder visit. Future work should compare patient-reported outcomes across patient groups to assess whether more expensive and invasive treatments yield better outcomes for patients with shoulder OA.

## Background

The shoulder is the third most common large joint affected by the degenerative condition osteoarthritis (OA) [1], and OA of the shoulder may affect as many as one-third of patients over the age of 60 [2]. Shoulder OA is associated with significant pain and reduction in mobility and quality of life [2, 3], yet treatment for shoulder OA is not definitive and includes both conservative and surgical modalities [2]. Current recommendations favor conservative management as initial treatment for shoulder OA [4]. Patients with symptomatic shoulder OA can choose from a wide range of physicians to treat their condition, and patients may initially visit a primary care practitioner or a specialist such as an orthopaedic surgeon to begin their treatment. There is no consensus on the optimal provider to initiate care and the specialty of the first provider contact for OA may shape each patient’s treatment course [5–7].

Shoulder OA is a common orthopaedic complaint in primary care medicine [8–10]. Yet, research suggests that primary care physicians receive limited training in musculoskeletal diseases [11, 12] compared to orthopaedic specialists who receive comprehensive training on the diagnosis and treatment of complex musculoskeletal conditions [13]. The healthcare literature lacks consensus as to the type of physicians who should care for patients with particular medical conditions [14, 15]. Specialists have been shown to achieve better clinical outcomes for some conditions such as myocardial infarction, stroke, asthma, and rheumatoid arthritis [16–22]. In most cases specialists know more about [22, 23] and are more likely to use optimal treatments their areas of expertise [24]. However, at times the treatment regimens provided by specialists have been shown to be more expensive and wasteful [16, 19, 24–27]. While others have assessed the factors affecting treatment utilization post OA diagnosis for patients initiating care with orthopaedic specialists [28], this will be the first to compare orthopaedic treatment utilization for patients initiating care with non-orthopaedic physicians and orthopaedic specialists. We hypothesized that patients initially seeking care from an orthopaedic specialist would be more likely to receive invasive treatment such as surgical care and less likely to receive conservative treatment for shoulder OA.

## Methods

### Data sources and overview

Data for this study included standard billing records from 2012 to 2014 for patients diagnosed with shoulder OA in 2013 from a single large regional healthcare system. The health system where the study was performed is one of the largest integrated healthcare systems in the Southeastern US, with over 15,000 employees across 7 medical campuses and 155 affiliated practice sites. Standard billing records included service-line level information such as the date of service, billing physician, service facility, Current Procedural Terminology (CPT) and International Classification of Disease, Ninth Revision (ICD-9-CM) diagnostic codes associated with each healthcare service provided as well as patient age, sex and insurance status. These data were used for cohort identification and measurement of treatment utilization. In addition, medical charts were abstracted for a subset of the study sample. Medical chart data included clinical data not available in the standard billing records such as body mass index (BMI), smoking status, pain score and symptom duration. This study was approved by the Health System Institutional Review Board where the study was conducted (intentionally blinded).

### Patient sample

**Fig. 1:**
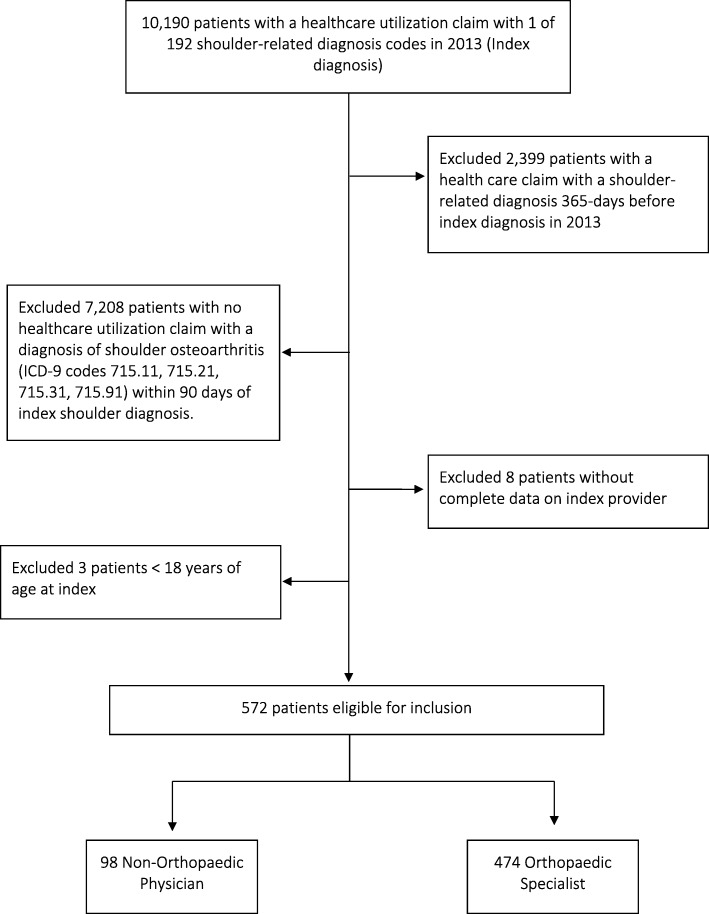
Derivation of the final sample used for analysis of patients seeking care for Shoulder Osteoarthritis

We identified all patients with an Evaluation and Management visit (E/M visit: CPT codes 992XX) in the health system that had at least 1 of 192 ICD-9-CM diagnosis codes related to shoulder pain or dysfunction in 2013. The date of the first visit with a shoulder-related diagnosis was designated, and is henceforth referred to as the index shoulder visit. Patients with any shoulder-related diagnosis, as defined above, in the period of 365 days prior to their index shoulder visit were excluded to allow researchers to make comparisons across patients seeking care for a new shoulder problem. Patients with shoulder OA were then identified as those with a diagnosis code from a clinical exam confirming shoulder OA in the period of 90-days after their index shoulder visit (ICD-9 codes 715.11, 715.21, 715.31, 715.91); all other patients without a diagnosis of shoulder OA were excluded. Patients who were less than 18-years old at index or who had incomplete data (e.g. patient age, gender, visit date, etc.) for creating study measures were excluded. The final cohort meeting all inclusion criteria included 572 patients. A patient sample flow chart is included in Fig. 1.

A retrospective chart review was conducted to compare patient and clinical variables that were not available in standard billing data. Due to inconsistencies in charting practices, clinical data such as pain scores and symptom duration were often missing from patient charts in the non-orthopaedic setting. Multiple rounds of stratified simple random sampling were used to identify and select complete patient charts for review. Only 24 out of 98 patient charts from non-orthopaedic physicians contained complete clinical information. Therefore, we selected and reviewed all 24 complete non-orthopaedic physician patient charts and selected a matched sample of 24 complete orthopaedic specialist patient charts to conduct the retrospective chart review.

### Measures

#### Physician specialty designation

The specialty of the physician or health care provider which was listed as the billing physician on the index shoulder visit for each patient was identified by linking providers to the National Plan and Provider Enumeration System files, which contain specialty information as taxonomy codes, by unique National Provider Identification (NPI) numbers [29]. Physician specialty was defined based upon the taxonomy code designated as most current. Physicians and health care providers (nurse practitioners) were then classified, based on specialty, as being either non-orthopaedic physicians (NOP) or orthopaedic specialists (OS). NOP included mainly family and internal medicine physicians (65.3%), rheumatologists (21.4%) and other non-orthopaedic specialties (13.3%). Other specialties included pain management specialists (7.1%), neurosurgeons (3.1%), physical medicine and rehabilitation specialists (2.0%) and general surgeons (1.0%). OS included orthopaedic surgeons (80.4%) and sports medicine trained primary care physicians (19.6%). Sports medicine primary care physicians were classified as OS because they are fellowship trained in musculoskeletal conditions and practice alongside orthopaedic surgeons in the local health system.

#### Treatment utilization variables

Treatments were grouped into four modalities and ranked in order of invasiveness. The hierarchy of invasiveness was established through clinical discussion with a practicing physical therapist and was assessed through evaluation of treatment time and potential complications. Physical therapy was considered the lowest level of treatment, followed by corticosteroid injections, arthroscopic surgery and finally total joint replacement. Treatment consisted of four separate modalities, defined as follows:

1. Physical therapy (CPT code: 29240, 76,881 76,942, 970XX, 971XX, 975XX)
2. Corticosteroid injections (CPT code: 205XX, 206XX, J3301, J0702)
3. Arthroscopic surgery (CPT code: 298XX, 23,020, 23,130, 23,430, 23,700)
4. Total joint replacement (CPT code: 23470, 23,472)

The treatment period was defined as 365-days following the index shoulder visit. Patients receiving no treatment during the treatment period were classified in a period of watchful waiting. Time to OA diagnosis from index shoulder visit and time from index shoulder visit to first treatment received were assessed for each patient, and measured in days. The first treatment modality received by each patient, if a treatment modality was ever received, and the number of physical therapy (PT) sessions and injections received during the treatment period were assessed for each patient. The first treatment received was defined as the first treatment received after the index shoulder visit. If multiple treatment modalities were used on the same date, the first treatment received was recorded as the most invasive treatment modality of those used on the same day. If more than one treatment was received throughout the treatment period; both treatment modalities were recoded and included in the analysis of treatments ever received. We assessed differences in treatment utilization variables between patients grouped by the provider specialty of their index shoulder visit.

#### Covariates

It is well established that comorbidity burden is a patient factor that is expected to influence treatment choices and treatment outcomes for patients [30, 31]. To control for differences in comorbidity burden across patients at index, billing data were used to assess healthcare utilization in the 365 days prior to the index shoulder visit for measures of baseline patient health. General comorbidity burden was measured using the Charlson Comorbidity Index (CCI) [32]. CCI is a validated measure of burden of disease. Comorbidities are weighted from 1 to 6 for mortality risk and disease severity, and then summed to form the total CCI score. Additionally, the number and type of healthcare visits by type (e.g. non-orthopaedic physician visits and orthopaedic specialist visits) in the year preceding the index shoulder visit were measured under the theory that higher use reflects poorer health status. Shoulder-specific health was assessed using concomitant shoulder diagnoses received within 90 days following the index shoulder visit.

Medical chart data extraction was performed by a team of two medical students. Patient charts from the index shoulder visit were reviewed and clinical data including body mass index (BMI), smoking status, pain score and symptom length, were extracted and recorded for each patient.

### Analyses

Patient characteristics at baseline and treatment utilization were compared between patient groups. Conservative baseline patient comparisons were based on 95% confidence intervals. The Shapiro Wilk test was used to assess normality of continuous variables. Treatment utilization was compared between patient groups using the Wilcoxon rank-sum test for continuous variables and Pearson’s chi-square and Fisher’s exact test for categorical data. Significance was established at *p* < 0.05. Multivariable logistic regression was used to estimate the independent influence of specialty of first provider seen and probability of receiving surgical treatment for shoulder OA. Models were adjusted for patient’s age, gender, insurance type, previous healthcare utilization, and concurrent shoulder diagnoses. The primary independent variable, specialty of first provider, was modeled as a dichotomous variable (1 = OS, 0 = NOP). Patient age was modeled as dummy variable with age categories of 18–34, 35–49, 50–64, 65–79 and 80 and above. Patient sex was a dichotomous variable of 1 = male and 0 = female. Insurance status was modeled as a dummy variable for public, private, other insurance and workers compensation. Previous health care visits and shoulder diagnoses were included as dichotomous variables (1 = yes, 0 = no). Concurrent shoulder diagnoses were specified in the model using two variables: one indicating whether the patient had any diagnosis of rotator cuff tear within 90 days following the index shoulder visit and another indicating whether the patient had any diagnosis of rheumatoid arthritis, humerus fracture, adhesive capsulitis or instability. The grouping of these conditions was based on their near-zero variance, each was present in less than 4% of the sample. Results are presented as adjusted Odds Ratios (OR) with accompanying 95% confidence intervals (95% CI). SAS software (Version 9.4) and R (Version 1.0.153) were used for data cleaning and statistical analyses.

## Results

### Study sample by physician specialty

Of the 572 patients included in the study, 474 (83%) were provided care from an OS on the date of index shoulder visit, while 98 (17%) initiated care with a NOP (Table 1). Patients in the study ranged from 20 to 95 years of age. There was no difference in the mean age or proportion of male patients initiating care with an OS compared to a NOP. A larger proportion of patients initiating care with an OS had a concurrent diagnosis of rotator cuff tear (33.1% [95% CI, 28.9, 37.6] vs. 16.3% [95% CI, 9.6, 25.2]) and a smaller proportion had a concurrent diagnosis of a chronic joint problem requiring ongoing management, such as rheumatoid arthritis (0.0% [95% CI, 0.0, 1.2] vs. 7.1% [95% CI, 2.9, 14.2]), compared to patients initiating care with an NOP. A higher proportion of OS patients were publicly insured (55.3% [95% CI, 50.7, 59.8] vs. 45.9% [95% CI, 35.8, 56.3]) or had a worker’s compensation claim (7.0% [95% CI, 4.8, 9.6] vs. 2.0% [95% CI, 0.2, 7.2]), compared to NOP patients. There were no meaningful differences in Charlson Comorbidity Index scores among NOP and OS patients, although a larger proportion of patients of NOP visited a NOP in the year prior to their index shoulder visit (71.4% [95% CI, 61.4, 80.1] vs 31.9% [95% CI, 27.7, 36.3]).

**Table 1:** Measures of shoulder health and general health from billing data for shoulder osteoarthritis patients by physician specialty (*N* = 572)

*Patient Characteristics*		Total Study Sample (*N* = 572)	Non-Orthopaedic Physician (*N* = 98)	Orthopaedic Specialist (*N* = 474)
Age	Mean	60.3	61.5	60.1
	(95% CI)	(59.2, 61.4)	(58.9, 64.1)	(58.9, 61.3)
Male Sex	n (%)	302 (52.8)	44 (44.9)	258 (54.4)
	(95% CI)	(48.6, 56.9)	(34.8, 55.3)	(49.8, 59.0)
Insurance type				
Public	n (%)	307 (53.7)	45 (45.9)	262 (55.3)
	(95% CI)	(49.5, 57.8)	(35.8, 56.3)	(50.7, 59.8)
Private	n (%)	219 (38.3)	48 (48.9)	171 (36.1)
	(95% CI)	(34.3, 42.4)	(38.7, 59.3)	(31.7, 40.6)
Worker’s compensation	n (%)	35 (6.1)	2 (2.0)	33 (7.0)
	(95% CI)	(4.3, 8.4)	(0.2, 7.2)	(4.8, 9.6)
Other	n (%)	11 (1.9)	3 (3.1)	8 (1.7)
	(95% CI)	(0.9, 3.4)	(0.6, 8.7)	(0.7, 3.3)
Concurrent Shoulder Diagnoses^a^				
Rotator Cuff Tear	n (%)	173 (30.2)	16 (16.3)	157 (33.1)
	(95% CI)	(26.5, 34.2)	(9.6, 25.2)	(28.9, 37.6)
Rheumatoid Arthritis	n (%)	7 (1.2)	7 (7.1)	0 (0.0)
	(95% CI)	(0.6, 2.7	(2.9, 14.2)	(0.0, 1.2)
Humerus Fracture	n (%)	6 (1.0)	0 (0.0)	6 (1.3)
	(95% CI)	(0.4, 2.3)	(0.0, 3.7)	(0.5, 2.7)
Instability	n (%)	9 (1.6)	0 (0.0)	9 (1.9)
	(95% CI)	(0.7, 3.0)	(0.0, 3.7)	(0.9, 3.6)
Adhesive capsulitis	n (%)	19 (3.3)	1 (1.0)	18 (3.8)
	(95% CI)	(2.0, 5.1)	(0.03, 5.5)	(2.2, 5.9)
Charlson Comorbidity Index^b^				
Score 0	n (%)	223 (69.0)	55 (68.0)	168 (69.4)
	(95% CI)	(63.7, 74.0)	(56.6, 77.8)	(63.2, 75.2)
Score 1	n (%)	54 (16.7)	15 (18.5)	39 (16.1)
	(95% CI)	(12.8, 21.2)	(10.7, 28.7)	(11.7, 21.4)
Score 2	n (%)	38 (11.8)	7 (8.6)	31 (12.8)
	(95% CI)	(8.5, 15.8)	(3.5, 17.0)	(8.9, 17.7)
Score 3	n (%)	4 (1.2)	2 (2.5)	2 (0.8)
	(95% CI)	(0.3, 3.1)	(0.3, 8.6)	(0.1, 2.9)
Score 4	n (%)	2 (0.6)	1 (1.2)	1 (0.4)
	(95% CI)	(0.08, 2.2)	(0.03, 6.7)	(0.01, 2.3)
Healthcare utilization in the 365 days prior to index shoulder visit				
Patients who visited a NOP	n (%)	221 (38.6)	70 (71.4)	151 (31.9)
	(95% CI)	(34.6, 42.7)	(61.4, 80.1)	(27.7, 36.3)
Number of visits to NOP^c^	Mean	4.1	4.3	4.0
	(95% CI)	(3.6, 4.7)	(3.6, 5.0)	(3.3, 4.8)
Patients who visited an OS	n (%)	88 (15.4)	18 (18.4)	70 (14.8)
	(95% CI)	(12.5, 18.6)	(11.3, 27.5)	(11.7, 18.3)
Number of visits to OS^d^	Mean	2.2	2.1	2.3
	(95% CI)	(1.9, 2.6)	(1.5, 2.7)	(1.9, 2.7)

^a^Among all OA patients

^b^Among patients with healthcare utilization in previous 365 days

^c^Among patients having one or more visit with non-orthopaedic physician

^d^Among patients having one or more visit with an orthopaedic specialist

### Treatment utilization by physician specialty

Table 2 shows additional comparisons of treatment utilization by initiating physician group. There was no significant difference in the time from index shoulder visit to OA diagnosis across patients by specialty of the initiating physician. However, patients initiating care with an OS received their first treatment in significantly less time (16.3 days [95% CI, 12.8, 19.7] vs. 32.3 days [95% CI, 21.0, 43.6] Z = 4.9, *p* < 0.01) compared to patients initiating care with a NOP. Injection was the most common first treatment modality for both NOP (33.7% [95% CI, 24.4, 43.9]) and OS patients (53.4% [95% CI, 48.8, 57.9]). A significantly larger proportion of OS patients received arthroscopic surgery (15.2% [95% CI, 12.1, 18.7]) or total joint replacement (4.8% [95% CI, 3.1, 7.2]) as their first treatment modality compared to NOP patients, of whom 7.1% [95% CI, 2.9, 14.2] received arthroscopic surgery and 2.0% [95% CI, 0.2, 7.1] received total joint replacement as their first treatment (*p*-value < 0.001 by Fisher’s exact test).

**Table 2:** Treatment utilization for shoulder osteoarthritis patients by physician specialty (*N* = 572)

		Total Study Sample (*N* = 572)	Non-Orthopaedic Physician (*n* = 98)	Orthopaedic Specialist (*n* = 474)	*p* value
Days from index shoulder visit to OA diagnosis	Mean	19.6	21.6	19.2	0.25*
	(95% CI)	(17.5, 21.8)	(16.3, 26.9)	(16.9, 21.6)	
Days from index shoulder visit to first treatment	Mean	18.2	32.3	16.3	< 0.01*
	(95% CI)	(14.8, 21.5)	(21.0, 43.6)	(12.8, 19.7)	
First Treatment Initiated					
Physical therapy	n (%)	66 (11.5)	13 (13.3)	53 (11.2)	< 0.01^‡^
	(95% CI)		(7.3, 21.6)	(8.5, 14.4)	
Injection	n (%)	286 (50.0)	33 (33.7)	253 (53.4)	
	(95% CI)		(24.4, 43.9)	(48.8, 57.9)	
Arthroscopic surgery	n (%)	79 (13.8)	7 (7.1)	72 (15.2)	
	(95% CI)		(2.9, 14.2)	(12.1, 18.7)	
Total joint replacement	n (%)	25 (4.4)	2 (2.0)	23 (4.8)	
	(95% CI)		(0.2, 7.1)	(3.1, 7.2)	
Watchful Waiting	n (%)	116 (20.3)	43 (43.9)	73 (15.4)	
	(95% CI)		(33.9, 54.3)	(12.3, 19.0)	
Treatment ever received^a^					
Physical therapy	n (%)	94 (16.4)	21 (21.4)	73 (15.4)	0.14^†^
	(95% CI)		(13.8, 30.9)	(12.3, 19.0)	
Injection	n (%)	351 (61.4)	46 (46.9)	305 (64.3)	< 0.01^†^
	(95% CI)		(36.8, 57.3)	(59.8, 68.7)	
Arthroscopic surgery	n (%)	196 (34.3)	18 (18.4)	178 (37.5)	< 0.01^†^
	(95% CI)		(11.3, 27.5)	(33.2, 42.1)	
Total joint replacement	n (%)	52 (9.1)	4 (4.1)	48 (10.1)	0.08^‡^
	(95% CI)		(1.1, 10.1)	(7.5, 13.2)	
Watchful Waiting	n (%)	116 (20.3)	43 (43.8)	73 (15.4)	< 0.01^†^
	(95% CI)		(33.9, 54.3)	(12.3, 19.0)	
Number of PT sessions over treatment period^c^	Mean	1.2	1.2	1.2	0.81*
	(95% CI)	(1.1, 1.3)	(1.0, 1.4)	(1.1, 1.3)	
Number of injections over treatment period^b^	Mean	1.6	1.6	1.6	0.42*
	(95% CI)	(1.5, 1.7)	(1.4, 1.9)	(1.5, 1.7)	
Days from index shoulder visit to surgery^d^	Mean	65.4	96.9	62.5	< 0.01*
	(95% CI)	(58.0, 72.8)	(62.6, 131.1)	(55.1, 69.8)	

^a^Groups are not mutually exclusive and will sum to greater than 100 %

^b^Among patients receiving at least one injection in the treatment period

^c^Among patients receiving at least one physical therapy session in the treatment period

^d^Among patients receiving arthroscopic surgery or total joint replacement in the treatment period

*p-value produced from Wilcoxon rank-sum test

^†^*p*-value produced from Pearson’s chi-square test

^‡^*p*-value produced from Fisher’s exact test

During the 365-day treatment period, 64.3% [95% CI, 59.8, 68.7] of patients initiating care with OS and 46.9% [95% CI, 36.8, 57.3] of those initiating care with NOP received injections (*X*^*2*^ (1, *N* = 572) = 10.4, *p*-value < 0.01). A larger proportion of NOP patients utilized physical therapy (21.4% [95% CI, 13.8, 30.9]) than OS patients (15.4% [95% CI: 12.3, 19.0]; *X*^*2*^ (1, N = 572) = 2.1, p-value 0.14), but there was no significant difference in the average number of physical therapy visits (1.2 PT visits [95% CI, 1.0, 1.4] for NOP; 1.2 PT visits [95% CI, 1.1, 1.3] for OS) or injections (1.6 injections [95% CI, 1.4, 1.9] for NOP; 1.6 injections [95% CI, 1.5, 1.7] for OS) received across groups. Thirty-seven percent [95% CI, 33.2, 42.1] of orthopaedic patients received arthroscopic surgery at some time during the treatment period, compared to only 18.4% [95% CI, 11.3, 27.5] of non-orthopaedic patients (*X*^*2*^ (1, *N* = 572) = 13.3, *p*-value< 0.01). Among the 43% (*N* = 248) of patients receiving arthroscopic or arthroplasty surgery during the treatment period, patients initiating care with an OS received surgical treatment in significantly less time (62.5 days [95% CI, 55.1, 69.8] vs. 96.9 days [95% CI, 62.6, 131.1] Z = 2.9, *p* < 0.01) than NOP patients. Forty-three percent [95% CI, 33.9, 54.3] of NOP patients received none of the specified treatments during the 365-day treatment period, compared to only 15.4% [95% CI, 12.3, 19.0] of patients initiating care with an OS (*X*^*2*^ (1, *N* = 572) = 40.7, *p*-value < 0.01).

Table 3 shows results from a logistic regression model predicting surgical treatment in the year following index shoulder visit. The adjusted odds of surgery were significantly higher for patients visiting an orthopaedic specialist on their index shoulder visit (OR = 2.65 [95% CI, 1.42, 4.95]) compared to non-orthopaedic patients.

**Table 3:** Adjusted odds ratios of surgical treatment (*N* = 572)

	Model
	Surgery within 1 year^±^
Index visit with Non-Orthopaedic Physician	*Reference*
Index visit with Orthopaedic Specialist	2.65** [1.42, 4.95]
Male	1.40 [0.93, 2.11]
Age	
18–34	*Reference*
35–49	1.00 [0.27, 3.62]
50–64	0.72 [0.20, 2.57]
65–79	0.44 [0.12, 1.70]
80+	0.18* [0.04, 0.92]
Insurance Type	
Private	*Reference*
Public	1.08 [0.61, 1.90]
Other	1.45 [0.34, 6.25]
Worker’s Comp	2.54* [1.05, 6.17]
Previous Healthcare Utilization	
Family Medicine visit	0.94 [0.61, 1.46]
Orthopaedic Specialist visit	1.35 [0.76, 2.39]
Concurrent Shoulder Diagnoses	
Rotator Cuff Tear	10.69*** [6.76, 16.93]
Other shoulder diagnosis	2.16* [0.99, 4.71]

Exponentiated coefficients interpreted as Odds Ratio; 95% confidence interval in parentheses

^±^Total joint replacement or arthroscopic surgery received within 1-year of index shoulder visit

^+^*p* < .1, *p < .05, **p < .01, ***p < .001

### Comparison of key variables from charts

Complete detail on characteristics of the chart abstraction sample is provided in Table 4.

**Table 4:** Clinical characteristics from retrospective chart review for shoulder osteoarthritis patients by physician specialty (*N* = 48)

		Total Study Sample (*N* = 48)	Non-Orthopaedic Physician (*n* = 24)	Orthopaedic Specialist (*n* = 24)
BMI	Mean	34.4	36.3	32.6
	(95% CI)	(30.1, 38.8)	(28.1, 44.5)	(30.0, 36.2)
Number of current smokers	n (%)	5 (10.4)	2 (8.3)	3 (12.5)
	(95% CI)	(3.5, 22.7)	(1.0, 27.0)	(2.7, 32.4)
Symptom duration in months	Mean	10.7	3.5	17.9
	(95% CI)	(5.4, 16.0)	(0.3, 6.8)	(8.3, 27.5)
Pain scale score	Mean	5.7	5.7	5.7
	(95% CI)	(5.0, 6.4)	(4.4, 7.0)	(4.8, 6.6)

Patients initiating care with an OS reported longer symptom duration (17.9 months [95% CI, 8.3, 27.5]) compared to patients initiating care with NOP (3.5 months [95% CI, 0.3, 6.8]). However, there was no difference in the BMI, pain score, or proportion of smokers at the index visit between patient groups.

## Discussion

To our knowledge, this study is the first to investigate the relationship between a patient’s initial provider choice and their orthopaedic treatment utilization in the year following a shoulder diagnosis. Results from this study show that patients initially seeing an OS for OA have a higher proportion of concurrent shoulder diagnoses and report longer symptom duration than NOP patients, although they do not differ in Charlson comorbidity index scores, age, sex, BMI, smoking status or pain scores. Across patient groups, time from initial shoulder visit to diagnosis of OA was not clinically or statistically different. However, patients initiating care with an OS received their first treatment on average much faster and were more likely to receive surgery in the year following their index shoulder visit than patients initiating care with a NOP.

Our findings suggest there are clear differences in shoulder OA treatment utilization for patients initially receiving care from NOP and OS. The shorter time to treatment suggests that patients may receive more immediate symptom relief if care is initiated with an OS. Additionally, a larger proportion of OS patients received surgical treatment, including arthroscopic surgery and total joint replacement compared to patients seeing a NOP. Patients of OS did report longer symptom duration and more concurrent shoulder diagnoses, suggesting that their overall shoulder health may be more severe than that of patients seeing a NOP.

While our results show differences in treatment utilization across patients initiating care with different physician specialties, they do not provide evidence as to appropriateness of care or which type of physician provided “better” care for shoulder OA. Our study can’t conclude whether higher use of surgical treatment resulted in improved patient outcomes for patients receiving care from OS. Although, in a study among patients with shoulder pain, Kuijpers and colleagues found that patients reporting persistent symptoms generated more than twice as much costs compared to patients reporting recovery after 6 months [33]. This supports the theory that early intervention, if effective in slowing the disease progression or removing the degenerative bone and cartilage, may eliminate the need for ongoing shoulder treatment. Furthermore, chronic shoulder pain lasting longer than 3 months has been shown to increase depression, anxiety and sleep disruptions [34]. Therefore, early, effective treatment may have wide ranging positive effects on a patient’s physical as well as mental health. Inference on effectiveness of early and more aggressive treatment paths can best be assessed with information on long-term orthopaedic treatment utilization and patient-reported outcomes. Future work needs to compare patient-reported outcomes across physician and treatment groups to more completely answer questions surrounding comparative risks and benefits of receiving care from OS versus other provider types. Furthermore, given the heterogeneity of treatment effects across patients, the observed variation in treatment across patients and entry points may reflect the effective mix for this population and treatments are properly distributed across patients.

Several important limitations of this study must be acknowledged more completely. The health system in which the study was conducted has a well-known orthopaedic practice led by a well-known shoulder specialist. It is possible that the proportion of patients seeking care from OS is higher than might be expected elsewhere and the treatment courses observed may also be unique to the health system. Moreover, because the data used for this study comes from a single healthcare system, it is possible that patients in the sample received care from other outside providers that we do not observe which could result in misclassification bias. It is possible that patients may have visited a NOP provider and been referred to an OS provider. This would result in the more severe shoulder cases appearing in the OS provider group. However, the health system where the study was performed is one of the largest integrated healthcare systems in the Southeastern US which increases the likelihood that we captured a patient’s first touch with the healthcare system. In efforts to ensure we captured the first shoulder visit, we included shoulder diagnosis codes which occurred in any diagnosis position. To confirm our findings we would recommend a larger study be conducted across many healthcare sites and systems. Similarly, administrative billing records did not contain pharmacologic treatment information, so we could not include pain medication utilization in our results. Unfortunately, due to inconsistent radiologic documentation we could not assess the stage of OA for each patient. However, because there were no observed differences in age or pain scale across patients, we do not expect meaningful differences in OA stage across patient groups.

We acknowledge that our sampling strategy for the selection of patient charts could potentially result in patient samples which were not representative of the larger patient populations of each physician group. Unfortunately we were limited by the number of charts which contained complete clinical information in the NOS setting and it is possible that patients with complete charts were systematically different than those without complete charts, and therefore are not representative of the larger NOS population. Furthermore, a sample of more than 24 charts should be reviewed from the larger OS group to ensure the sample is not biased. In order to confirm our results, a larger chart review should be conducted. If the patient populations do indeed differ in meaningful ways, the differences in treatment utilization we observed may be justified. We selected a parsimonious model for our logistic regression analysis; however we did not have patient information on underlying condition severity, patient socioeconomic status, or granularity surrounding patient insurance structure which may affect referral patterns. Lastly, it is possible that differences in treatment utilization were due to differences in patient preferences for treatment. More work is needed to compare patient preferences for treatment across provider types.

## Conclusions

This study is the first to explore differences in shoulder OA treatment utilization for patients who enter the healthcare system through different physician channels. Results show that patients initiating care with an OS received treatment faster and were treated with more invasive services over the year following their index shoulder visit. However, this study did not assess the effectiveness or appropriateness of different treatment utilization. Future work should compare patient-reported outcomes across physician and treatment groups in larger patient samples which contain multiple health systems.

## Abbreviations

BMI: Body Mass Index
CCI: Charlson Comorbidity Index
CPT: Current Procedural Terminology
E/M: Evaluation and Management
ICD-9-CM: International Classification of Disease, Ninth Revision
NOP: Non-orthopaedic physician
NPI: National Provider Identification
OA: Osteoarthritis
OS: Orthopaedic specialist
PT: Physical Therapy
